# Validation of a computerized model for a new biomechanical concept- the fossa-foveolar mismatch- the answer to lesions of the ligamentous fossa-foveolar complex in the hip?

**DOI:** 10.1007/s00402-024-05508-x

**Published:** 2024-09-23

**Authors:** Vera M. Stetzelberger, Jannine T. Segessenmann, Cem Cek, Vlad Popa, Joseph M. Schwab, Corinne A. Zurmühle, Alexander F. Heimann, Moritz Tannast

**Affiliations:** Department of Orthopaedic Surgery and Traumatology, HFR Cantonal Hospital, University of Fribourg, Fribourg, 1708 Switzerland

**Keywords:** Fossa-foveolar mismatch, Hip preserving surgery, Ligamentum teres, Hip pain, Acetabular fossa, Fovea capitis, Intraclass correlation coefficient, Range of motion, Hip morphology, Hip, Active patients, Validation, Biomechanical, 3D model, CT

## Abstract

**Background:**

Hip-preserving surgery in young patients frequently reveals lesions of the ligamentum teres (LT). Histological and clinical evidence supports that those lesions could be source of intraarticular hip pain. It has been hypothesized that LT degeneration could be linked to the abnormal positioning of the fovea outside the lunate surface during various daily motions. We introduce the “fossa-foveolar mismatch” (FFM) by determining the trajectory of the fovea in the fossa during hip motions, enabling a comparison across diverse hip-pathomorphologies. Aims: to determine (1) intraobserver reliability and (2) interobserver reproducibility of our computer-assisted 3-dimensional (3D) model of the FFM.

**Materials and methods:**

All patients with joint preserving surgery for femoroacetabular impingement syndrome (FAIS) or developmental dysplasia of the hip (DDH) at our institution (11. 2015–08.2019)were initially eligible. We employed a simple random sampling technique to select 15 patients for analysis. Three-dimensional surface models based on preoperative computed tomography (CT) scans were built, the fossa virtually excised, the fovea capitis marked. Models were subjected to physiological range of motion with validated 3D collision detection software. Using a standardized medial view on the resected fossa and the transparent lunate surface, the FFM-index was calculated for 17 motions. It was obtained by dividing the surface occupied by the fovea outside of the fossa by the total foveolar tracking surface. Three observers independently performed all analyses twice. (1) Intraobserver reliability and (2) interobserver reproducibility were calculated using intraclass correlation coefficients (ICCs).

**Results:**

(1) We obtained excellent intraobserver ICCs for the FFM-index averaging 0.92 with 95% CI 0.77–0.9 among the three raters for all motions. (2) Interobserver reproducibility between raters was good to excellent, ranging from 0.76 to 0.98.

**Conclusions:**

The FFM-index showed excellent intraobserver reliability and interobserver reproducibility for all motions. This innovative approach deepens our understanding of biomechanical implications, providing valuable insights for identifying patient populations at risk.

## Introduction

In young patients undergoing hip-preserving surgery, lesions of the ligamentum teres (LT) are common [3, 14]. While the precise biomechanical role of this ligament remains uncertain, it is widely acknowledged that such injuries result in persistent and challenging hip pain [2]. This is supported by a prior histological study that identified nerve endings within the LT [7]. Nevertheless, the exact mechanisms causing these injuries are not yet fully understood.

Klaue et al. [5] first proposed a potential explanation for LT degeneration, which involves the fovea capitis not being ideally positioned on the femoral head. In a three-dimensional (3D) study focused on surgical planning for dysplasia correction, they observed a more cranial positioning of the LT’s insertion on the fovea capitis in dysplastic hips. They postulated that during range of motion (ROM), the ligament could extend beyond the acetabular fossa and onto the articular surface, coining the term “fossa-fovea overlapping impingement.” However, to date, a comprehensive and quantitative analysis of this hypothesis has been absent from the scientific literature.

**Fig. 1:**
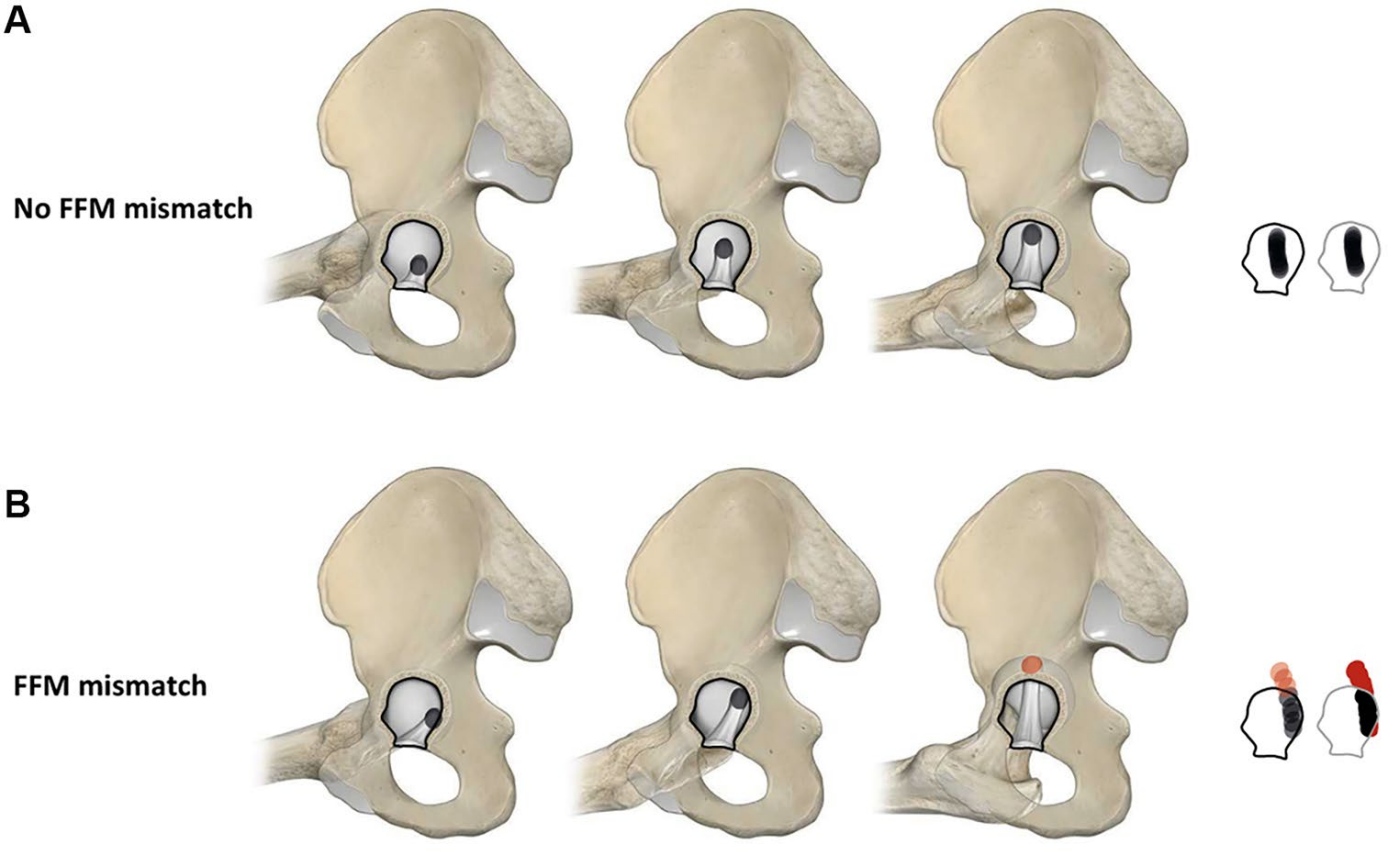
The fossa-foveolar mismatch is illustrated by displaying (**A**) no fossa-foveolar mismatch and (**B**) presence of a fossa-foveolar mismatch with a pathologic foveolar tracking pattern for the motion of everyday life internal/external rotation in 90 degrees flexion

Utilizing state of the art technological resources, specifically advanced 3D motion simulations, we extend the boundaries of this theoretical concept, calling it the fossa-foveolar mismatch [18]. We utilize a computer-tomography-based 3D model, enabling the visualization of the fovea’s trajectory and picturing the foveolar tracking pattern during various hip motions. For the purposes of quantification and comparison, we introduce the “fossa-foveolar mismatch index.” The index is calculated by dividing the surface of the tracking pattern located outside the acetabular fossa by the total tracking pattern surface. This novel approach facilitates comparisons across diverse hip pathomorphologies, offering a more comprehensive insight into the biomechanical implications and potentially identifying patient populations at risk (Fig. 1). In this context the aims of the present study were to determine the (1) intraobserver reliability and (2) interobserver reproducibility of our computer-assisted 3D model of the fossa-foveolar mismatch.

## Materials and methods

The present diagnostic study validates the computerized modelling of the fossa-foveolar mismatch on 15 patients from our institutional database. It was approved by our local institutional review board (KEK Bern, 2018-00078).

### Patients

**Fig. 2:**
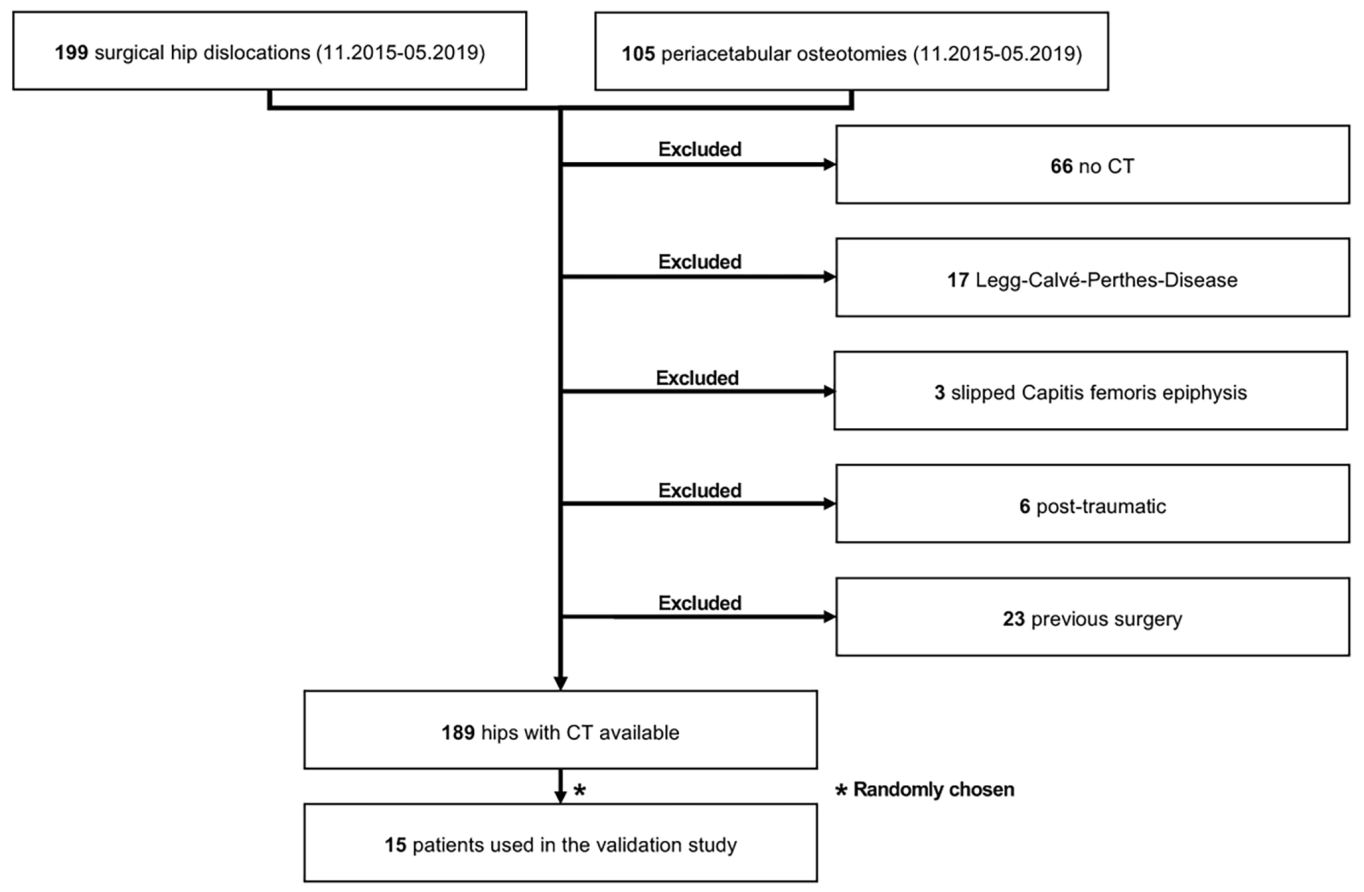
The flow diagram displays the inclusion and exclusion criteria of our study cohort

Among all patients with open hip preserving surgery for femoroacetabular impingement syndrome (FAIS) and developmental dysplasia of the hip (DDH) with osteoarthritis grade > Tönnis II (November 2015- May 2019) we excluded patients with significant acetabular and femoral pathomorphologies like Legg-Calvé-Perthese disease (LCPD) or slipped capital femoral epiphysis (SCFE), post-traumatic deformities, previous surgery or lack of computed tomography (CT) imaging (Fig. 2). Of the remaining hips, we employed a simple random sampling technique to select 15 patients. By using this approach, every patient in the database had an equal chance of being chosen for inclusion in the study. This method ensured that our sample was representative of the larger population of symptomatic patients who had undergone joint-preserving hip surgery at our institution. The mean age was 31 ± 10 (18–62), with 27% being male (Table 1).

**Table 1:** Demographic, radiographic, and surgical parameters of the patients

Category	Parameters	Value
Demography	Number of hips (patients)	15 (15)
	Age at surgery (years)	31 ± 10 (18–62)
	BMI, kg/m^2^	23 ± 3 (19–29)
	Male Sex (%)	4 (27)
	Right Side (%)	11 (73)
Diagnosis	Femoroacetabular Impingement, % total	15 (100)
	- Cam, %	3 (20)
	- Pincer, %	4 (27)
	- Torsional deformity, %	10 (67)
	- Associated dysplasia	7 (47)
Acetabular radiographic features	Lateral center edge angle, deg	27 ± 9 (14–42)
	Acetabular index, deg	7 ± 7 (-8–19)
	Extrusion index, deg	23 ± 9 (9–35)
	Anterior center edge angle, deg	37 ± 13 (17–61)
	Anterior acetabular coverage, %	21 ± 7 (0–30)
	Posterior acetabular coverage, %	44 ± 9 (32–66)
	Total acetabular coverage, %	75 ± 11 (59–93)
	Crossover sign, % positive	12 (80)
	Posterior wall sign, % positive	11 (73)
	Retroversion index, %	11 ± 13 (0–37)
	Ischial spine sign, % positive	9 (60)
Femoral radiographic features	Neck-shaft angle, deg	135 ± 6 (127–153)
	Alpha angle, deg	50 ± 8 (38–69)
	Femoral version, deg	33 ± 17 (0–68)

Continuous values are expressed as mean ± SD (range); other values are presented as number with percentage in parenthesis unless noted otherwise. BMI, body mass index

### Imaging

All patients underwent computed tomography (CT) scans including the entire pelvis and the distal femoral condyles [10, 11] according to a previously described protocol [12, 13]. Standardized anteroposterior (AP) pelvis and lateral hip radiographs were undertaken to assess acetabular and femoral radiographic parameters (Table 1). Acetabular morphology was categorized according to previously established reference values [16] on the AP pelvis radiograph. Femoral torsion was measured according to Murphy et al. [9].

### New concept: the fossa-foveolar mismatch

**Fig. 3:**
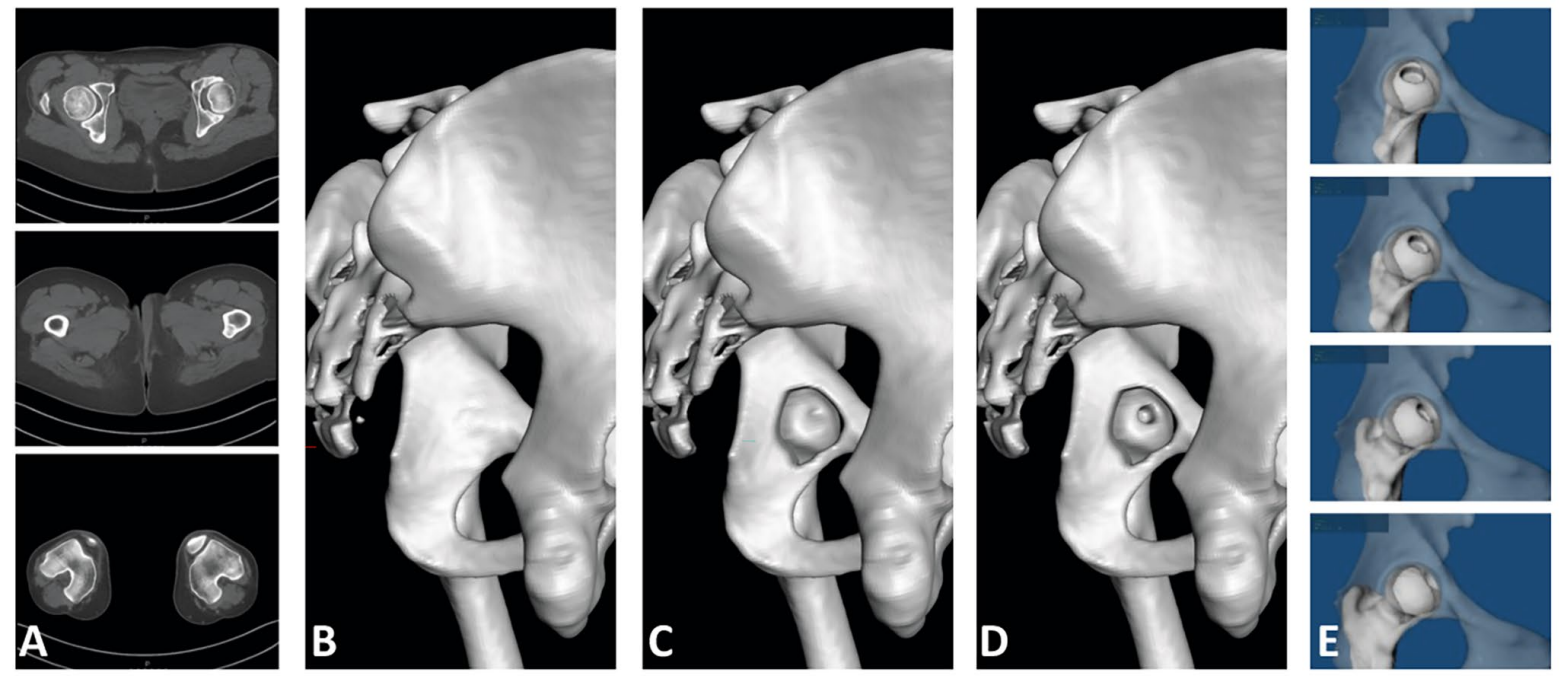
(**A**) Based on CT of the pelvis and distal femur; (**B**) we created a 3D model; (**C**) The acetabular fossa was digitally removed; (**D**) the fovea was identified and manually exaggerated; (**E**) finally, the model was subjected to standardized movements within physiological range of motion and the foveolar tracking pattern was determined

Based on each patient’s preoperative CT scan, we created a 3D surface model of the pelvis, proximal femur and distal femur using the semi-automatic segmentation software AMIRA (Thermo Fisher Scientific 2019.3, Waltham, MA USA; Fig. A-B). The inner part of the acetabulum was inspected thoroughly, and the area/shape of the acetabular fossa was identified. Then, the model was further edited by digital removal of the entire acetabular fossa using a specific software tool (Fig. 3C). On the femur, the shape of the fovea capitis was located, and, for reasons of visibility, its depth was manually increased by approximately 1 cm (Fig. 3D).

**Fig. 4:**
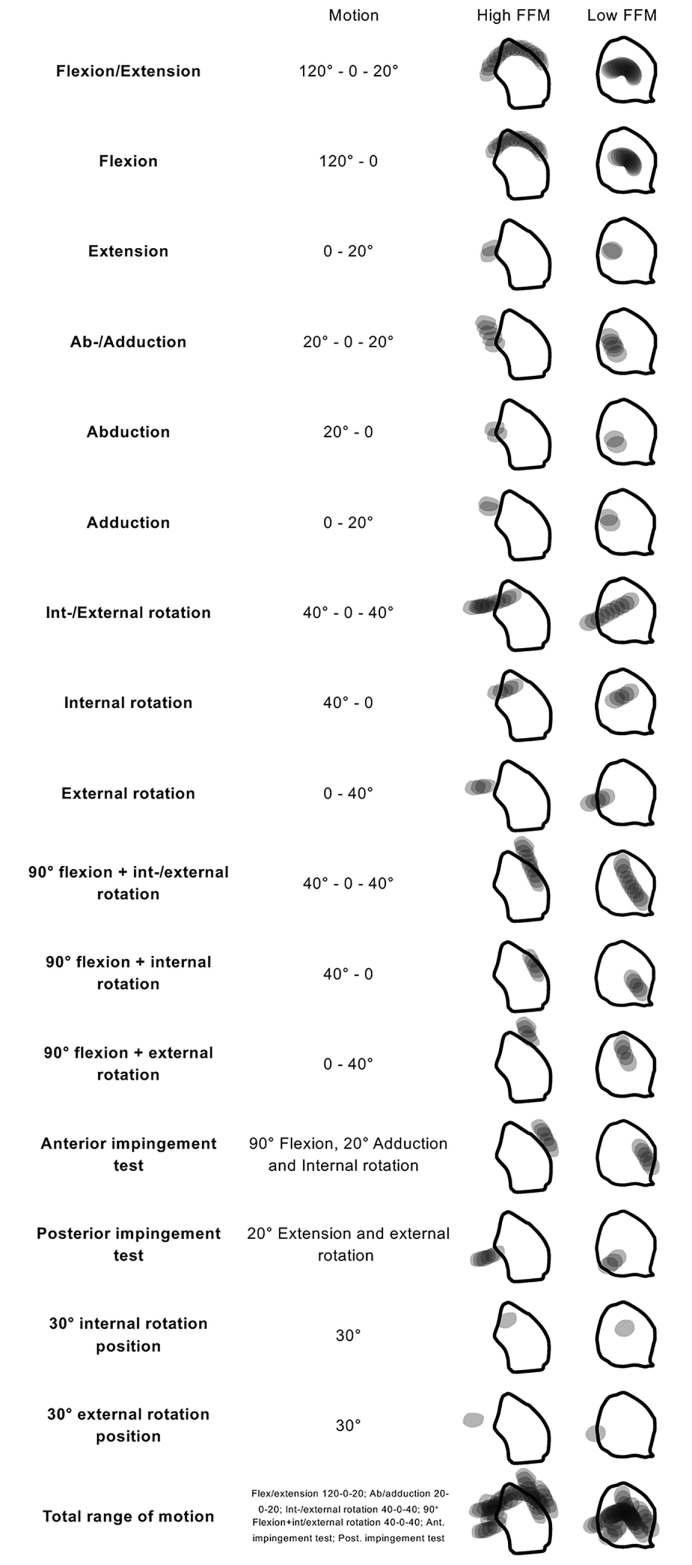
The foveolar tracking pattern for all analyzed motions is illustrated showing an example of a high and low FFM index

Using a previously validated and widely used 3D collision detection software [10, 15], we subjected every patient hip to the physiological range of motion [6]. Great attention was paid to the position of the joint during the simulation. The pelvis was placed in a specific orientation in which the origin of the transverse ligament at the acetabular fossa was horizontally aligned, and the femoral head was accurately positioned at the center of the acetabulum, ensuring a precise medial perspective of the femur’s motion within the acetabular fossa. By marking the positions of the fovea within the acetabular fossa, its tracking pattern was digitally assessed during all movements in 10° steps (Fig. 4). The following movements were assessed according to previously established values of physiological ROM: flexion/extension, ab/adduction, int/external rotation in 0° and 90° flexion, the anterior and posterior impingement test, and 30° int/external rotation positions. The surface of the tracking pattern located both within and outside the borders of the acetabular fossa were identified and the area of each quantified. The fossa-foveolar mismatch index was calculated (Table 2). Certain patients exhibited either intraarticular or extraarticular femoroacetabular impingement during end-range movements. We excluded positions that exceeded the point at which impingement was identified by our collision software. The surface of the tracking pattern located within and outside of the acetabular fossa were quantified. The fossa-foveolar mismatch index was calculated.

**Table 2:** Calculated values for the 15 patients

Analyzed motions for all patients (*n* = 15)	Fossa-foveolar mismatch (FFM) index
Flex-/extension index	0.09 ± 0.09 (0–0.27)
Flexion index	0.06 ± 0.08 (0–0.28)
Extension index	0.12 ± 0.16 (0–0.39)
Ab-/adduction index	0.16 ± 0.17 (0–0.54)
Abduction index	0.08 ± 0.14 (0–0.54)
Adduction index	0.17 ± 0.18 (0–0.54)
Int-/external rotation index	0.27 ± 0.09 (0.10–0.42)
Internal rotation index	0.09 ± 0.12 (0–0.42)
External rotation index	0.46 ± 0.23 (0.16–0.85)
90° flexion + int-/ external rotation index	0.19 ± 0.12 (0–0.39)
90° flexion + internal rotation index	0.09 ± 0.15 (0–0.57)
90° flexion + external rotation index	0.25 ± 0.18 (0–0.61)
Anterior impingement test index	0.41 ± 0.23 (0–0.84)
Posterior impingement test index	0.33 ± 0.20 (0–0.62)
30° internal rotation index	0.05 ± 0.13 (0–0.52)
30° external rotation index	0.49 ± 0.35 (0.09–0.98)
Total ROM index	0.34 ± 0.10 (0.12–0.52)

The entire procedure, encompassing the excision of the acetabular fossa, demarcation of the fovea capitis, and subsequent determination of the foveolar tracking pattern for each distinct motion, was executed by three observers (CC, JTS and VP, none of whom were treating surgeons). All observers conducted each measurement twice, with a minimum of two weeks in between. Using a total of 17 motion patterns described above in 15 patients analyzed by three observers twice, a total of 1530 measurements were available for analysis.

### Statistical analysis

We utilized intra-class correlation coefficients (ICC) with 95% confidence interval (CI) to determine the intraobserver reliability and interobserver reproducibility, defining values > 0.75 as excellent, 0.6–0.75 as good, 0.4–0.6 as fair and < 0.4 as poor [8].

## Results

### Intraobserver reliability

**Fig. 5:**
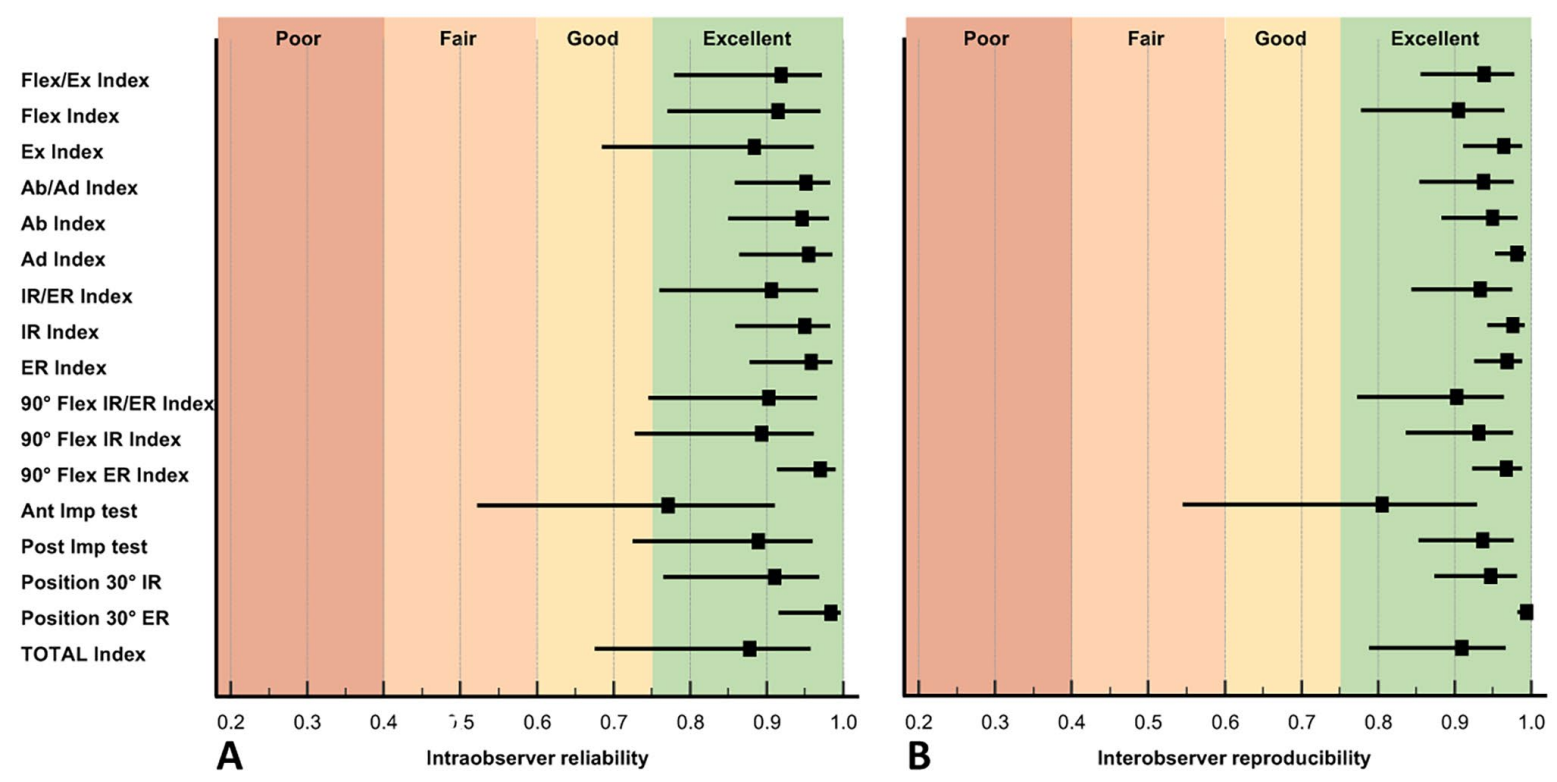
The forest plots show the (**A**) intraobserver reliability and (**B**) interobserver reproducibility of the FFM index for the different motions. Flex/Ex: Flexion/Extension; Ab/Ad: Abduction/Adduction; IR/ER: Internal/External rotation; 90 Flex IR/ER: Internal/External rotation in 90° flexion; Ant Imp test: Anterior Impingement test; Post Imp: Posterior Impingement test; Position 30° IR: FFM index in 30° internal rotation position; Position 30° ER: FFM index in 30° external rotation position;

The intraobserver reliability was excellent for all measured motions ranging from an average of 0.77 (95% CI 0.52 to 0.91) for the anterior impingement test to 0.97 (95% CI 0.91 to 0.99) for the motion of external rotation in 90° of flexion (Table 3; Fig. 5A).

**Table 3:** Results of the reliability and reproducibility analysis of the fossa foveolar mismatch indices in different motions

	Intraobserver reliability			Interobserver reproducibility	
	Observer 1 (CC)	Observer 2 (JS)	Observer 3 (VP)	First measurement	Second measurement
Flex-/extension index	0.92 (0.80 to 0.97)	0.90 (0.73 to 0.97)	0.93 (0.81 to 0.98)	0.95 (0.87 to 0.98)	0.93 (0.84 to 0.97
Flexion index	0.88 (0.69 to 0.96)	0.93 (0.80 to 0.98)	0.93 (0.82 to 0.98)	0.93 (0.84 to 0.97)	0.88 (0.72 to 0.96)
Extension index	0.93 (0.82 to 0.98)	0.80 (0.49 to 0.93)	0.91 (0.75 to 0.97)	0.97 (0.93 to 0.99)	0.96 (0.90 to 0.99)
Ab-/adduction index	0.96 (0.89 to 0.99)	0.95 (0.87 to 0.98)	0.94 (0.82 to 0.98)	0.93 (0.84 to 0.98)	0.94 (0.86 to 0.98)
Abduction index	0.93 (0.80 to 0.98)	0.95 (0.85 to 0.98)	0.96 (0.90 to 0.99)	0.95 (0.89 to 0.98)	0.95 (0.87 to 0.98)
Adduction index	0.97 (0.90 to 0.99)	0.95 (0.86 to 0.99)	0.94 (0.83 to 0.98)	0.99 (0.96 to 1.0)	0.98 (0.94 to 0.99)
Int-/external rotation index	0.96 (0.88 to 0.99)	0.95 (0.87 to 0.98)	0.81 (0.53 to 0.93)	0.93 (0.84 to 0.98)	0.93 (0.85 to 0.98)
Internal rotation index	0.91 (0.76 to 0.97)	0.96 (0.89 to 0.99)	0.98 (0.93 to 0.99)	0.98 (0.94 to 0.99)	0.98 (0.94 to 0.99)
External rotation index	0.94 (0.84 to 0.98)	0.97 (0.92 to 0.99)	0.96 (0.88 to 0.99)	0.96 (0.90 to 0.98)	0.98 (0.95 to 0.99)
90° flexion + int-/ external rotation index	0.96 (0.87 to 0.99)	0.81 (0.52 to 0.93)	0.94 (0.84 to 0.98)	0.86 (0.67 to 0.95)	0.95 (0.88 to 0.98)
90° flexion + internal rotation index	0.77 (0.44 to 0.91)	0.97 (0.89 to 0.99)	0.95 (0.86 to 0.98)	0.95 (0.89 to 0.98)	0.98 (0.94 to 0.99)
90° flexion + external rotation index	0.98 (0.93 to 0.99)	0.97 (0.92 to 0.99)	0.96 (0.89 to 0.99)	0.98 (0.94 to 0.99)	0.98 (0.95 to 0.99)
Ant. impingement test index	0.87 (0.67 to 0.96)	0.65 (0.00 to 0.88)	0.96 (0.89 to 0.99)	0.75 (0.42 to 0.91)	0.86 (0.67 to 0.95)
Post. impingement test index	0.75 (0.40 to 0.91)	0.96 (0.89 to 0.99)	0.96 (0.89 to 0.99)	0.91 (0.79 to 0.97)	0.97 (0.92 to 0.99)
30° internal rotation index	0.84 (0.60 to 0.94)	0.95 (0.85 to 0.98)	0.95 (0.85 to 0.98)	0.97 (0.93 to 0.99)	0.92 (0.82 to 0.97)
30° external rotation index	0.97 (0.88 to 0.99)	0.99 (0.91 to 1.00)	0.99 (0.69 to 1.00)	0.99 (0.98 to 1.0)	0.99 (0.98 to 1.0)
Total ROM index	0.92 (0.78 to 0.97)	0.84 (0.59 to 0.94)	0.87 (0.66 to 0.96)	0.90 (0.76 to 0.96)	0.92 (0.81 to 0.97)

Values are given as intraclass coefficients (ICC) with 95% confidence interval (CI) in parentheses

### Interobserver reproducibility

The interobserver reproducibility was excellent for all measured motions, ranging from an average between both measurements of 0.81 (95% CI 0.54 to 0.93) for the anterior impingement test to 0.98 (95% CI 0.95 to 0.99) for the motion of adduction (Table 3; Fig. 5B).

## Discussion

In the present study we introduce and validate a new concept we call the fossa-foveolar mismatch based on the initial description by Klaue et al. [5]. Although our approach uses a validated virtual dynamic hip motion algorithm, the definition of the fossa acetabuli, the location of the fovea capitis femoris, the anatomical reference landmarks for calculation of ROM, and the visual components of the index are manually determined. To apply this methodology to a larger number of subjects, an evaluation of the reliability and reproducibility is mandatory. We can assert that the methodology employed for calculating the FFM index is highly reliable and reproducible for all motions of the physiological range of motion. This is the first study aiming to quantify a long postulated potential pathomechanism for ligamentum teres tears.

### Limitations

The present validation study has limitations. First, the intended complete range of motion pattern could not be fully evaluated for all patients. Some patients presented an early intra- or extraaricular femoroacetabular impingement in terminal motion. We did not include the positions beyond the point of collision detection using our software model. This can theoretically underestimate the overlap of the fossa with the fovea but likely reflects the reality of the native hip without correction. Our approach would even allow to simulate the correction and potentially predict a mismatch after. Second, we analyzed osseous impingement motions only. In case of soft tissue contractures or constraints, the detected tracking pattern for the fossa-foveolar mismatch might have been overestimated. More specifically, it has been suggested that soft tissues could reduce a simulated range of motion by as much as 20° [1]. This could potentially implicate a systemic bias for the FFM-index values by influencing the absolute values. Nevertheless, this would not affect comparisons between motions of a same patient or pre- to postoperative values. Third, we quantified a three-dimensional problem using a two-dimensional projection. For us, this represents a very intuitive method of visualization. We used the exact same approach for all measurements, making the different tracking patterns comparable between different morphologies. However, it still may introduce bias into our assessment. Fourth, we use computer analysis only without clinical correlation. Since this represents a validation study of a method, making sure the reliability and reproducibility of the method is acceptable before correlating with a clinical picture is a logical first step. Last, the high percentage of female patients may apparently induce a bias. However, by using patients with cam, pincer, mixed femoroacetabular impingement (with or without femoral torsion abnormalities), as well as dysplasia, we validated the new concept for all pathomorphologies, regardless of the proportion of female patients.

#### Intraobserver reliability

Our findings indicate robust reliability across measurements conducted by three observers. Notably, no discernible motion-related systematic variations were observed. The anterior impingement motion, particularly in flexion, adduction, and internal rotation, demonstrated the lowest values. This trend can be attributed to several factors: firstly, the identification of early impingement during one measurement leads to a general reduction in the FFM index. Secondly, the anterior impingement test involves a tracking pattern with only four positions, in contrast to the more extensive 15 positions required for flexion/extension. Consequently, the presence or absence of individual tracking points has a more pronounced impact on the resulting FFM value. However, considering these nuances, our comprehensive results affirm the reliability of our methodology.

#### Interobserver reproducibility

Similarly to intraobserver reliability, we found promising values for interobserver reproducibility. The above-mentioned effect for the impingement motions also applies for this study variable. We could not identify a learning curve among the observers between the first and second measurement. The results confirm that manual detection of the size and shape of the acetabular fossa and the fovea are not subject to relevant individual observer-related variations. Thus, our methodology can be applied in a large cohort of patients.

When thoroughly analyzing the current literature, we could not find any comparable study providing data about observer-dependent differences in the setup of FFM including the initial description of the phenomenon. However, using computerized dynamic hip joint analysis, similar ICC values could be found for motion amplitudes [15] and femoral head coverage [17]. This underlines that such a computer approach is a valid and useful tool for analysis of central lesions of the hip joint in the setting of joint-preservation.

## Conclusion

Subsequent studies should concentrate on furnishing a comprehensive delineation of the FFM tracking pattern across distinct hip pathomorphologies, establishing correlations with intraoperative concomitant central lesions, and incorporating activity-specific motion patterns for an individual patients [4]. Our validated methodology serves as a springboard for further pursuit of this scientific inquiry.
